# Lysine-Less Variants of Spinal Muscular Atrophy SMN and SMNΔ7 Proteins Are Degraded by the Proteasome Pathway

**DOI:** 10.3390/ijms18122667

**Published:** 2017-12-08

**Authors:** Raúl Sánchez-Lanzas, José G. Castaño

**Affiliations:** Departamento de Bioquímica, Instituto de Investigaciones Biomédicas “Alberto Sols” (UAM-CSIC), Centro de Investigación Biomédica en Red sobre Enfermedades Neurodegenerativas (CIBERNED), Facultad de Medicina de la Universidad Autónoma de Madrid (UAM), 28029 Madrid, Spain; rslanzas@iib.uam.es

**Keywords:** survival motor neuron (SMN), ubiquitin, proteasome, protein degradation discipline

## Abstract

Spinal muscular atrophy is due to mutations affecting the *SMN1* gene coding for the full-length protein (survival motor neuron; SMN) and the *SMN2* gene that preferentially generates an exon 7-deleted protein (SMNΔ7) by alternative splicing. To study SMN and SMNΔ7 degradation in the cell, we have used tagged versions at the N- (Flag) or C-terminus (V5) of both proteins. Transfection of those constructs into HeLa cells and treatment with cycloheximide showed that those protein constructs were degraded. Proteasomal degradation usually requires prior lysine ubiquitylation. Surprisingly, lysine-less variants of both proteins tagged either at N- (Flag) or C-terminus (V5) were also degraded. The degradation of the endogenous SMN protein, and the protein constructs mentioned above, was mediated by the proteasome, as it was blocked by lactacystin, a specific and irreversible proteasomal inhibitor. The results obtained allowed us to conclude that SMN and SMNΔ7 proteasomal degradation did not absolutely require internal ubiquitylation nor N-terminal ubiquitylation (prevented by N-terminal tagging). While the above conclusions are firmly supported by the experimental data presented, we discuss and justify the need of deep proteomic techniques for the study of SMN complex components (orphan and bound) turn-over to understand the physiological relevant mechanisms of degradation of SMN and SMNΔ7 in the cell.

## 1. Introduction

Spinal muscular atrophy (SMA) by loss of lower motor neurons and atrophy of muscle is the leading genetic cause of infant mortality [1]. There are two SMN genes in the human genome, *SMN1* (telomeric) and *SMN2* (centromeric) located in chromosome 5q13 [2]. The *SMN1* gene encodes the full-length protein, while the *SMN2* gene preferentially generates an exon 7-deleted protein (SMNΔ7) by alternative splicing due to a C to T transition in the centromeric copy [3]. Both SMN and SMNΔ7 are degraded by the ubiquitin–proteasome pathway [4,5], with SMNΔ7 having a two-fold shorter half-life than SMN1 by radioactive pulse-chase experiments [5]. The SMNΔ7 has a new C-terminal (15 amino acids), and those amino acids were shown to destabilize other stable proteins when fused at their C-terminus [6]. UCHL1 (Ubiquitin C-terminal Hydrolase L1) has also been implicated in SMN protein degradation [7]. Confirming a previous description of the direct interaction of USP9X (Ubiquitin Specific Peptidase 9, X-Linked) with SMN [8], it was shown that USP9X knock-down promotes the degradation of Flag-tagged SMN constructs, but does not affect the degradation of Flag-SMN∆7 [9]. Finally, the E3-ligases. mind bomb 1 (Mib1), when overexpressed, interacts and ubiquitylates SMN [10] and Itchy E3 Ubiquitin Protein Ligase (Itch) has been implicated in the degradation of SMN protein [11].

A standard method to study the role of ubiquitylation in protein degradation uses tagging of the corresponding protein, but tags are not always neutral with respect to degradation [12]. As a consequence, tagged SMN and SMN∆7 at either their N- (Flag) or the C-terminus (V5) were constructed to study the ubiquitylation requirement for degradation. Hence, if usual ubiquitylation of Lys residues is required for degradation of SMN and SMN∆7, protein constructs in which all Lys residues were changed to Arg (Lys-less mutants, K0) should prevent its degradation. Unexpectedly, the K0 mutants of both SMN and SMN∆7 were still degraded by the proteasomal pathway. These results indicated that degradation of SMN and SMN∆7 does not absolutely require usual internal Lys or N-terminal ubiquitylation (or acetylation, see discussion) for degradation; but more importantly, they also question the suitability of the use of the tagged and mutant proteins for the study of the physiological relevant mechanisms of the degradation of SMN and SMN∆7.

## 2. Results

### 2.1. Endogenous SMN Degradation

**Figure 1:**
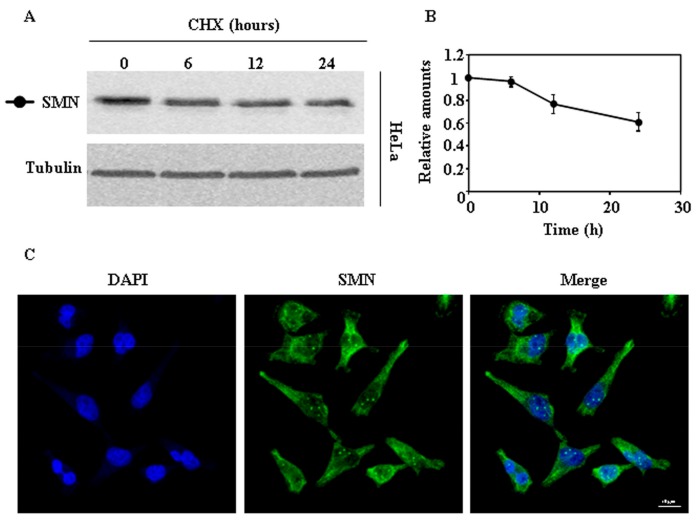
Degradation and subcellular localization of endogenous survival motor neuron (SMN) in HeLa cells. (**A**) HeLa cells were treated with cycloheximide (CHX) for the indicated times and cell extracts were analyzed by Western and immunoblot with anti-SMN antibodies. Anti-tubulin antibodies were used as total protein loading control. (**B**) Graph of quantification of the corresponding immunoblots. (**C**) Confocal fluorescence images of HeLa cells growing under basal conditions, stained with anti-SMN specific antibodies (green channel) and counterstained with DAPI (blue channel) for nuclei visualization. Bars = 10 µm. Data are mean ± S.E.M. from at least three different experiments.

We began the study of the degradation of endogenous SMN, as a reference, by treating HeLa cells with cycloheximide (CHX) and analyzing the time-course of decay by immunoblotting of total cellular extracts. As shown in Figure 1A, SMN was degraded in HeLa cells. Figure 1C shows the typical subcellular distribution, nuclear with some “foci” and cytoplasmic staining, of SMN as observed by confocal indirect immunofluoresecence of HeLa cells.

### 2.2. Tagging SMN and SMNΔ7 at Their N- or C-Terminus and Degradation

**Figure 2:**
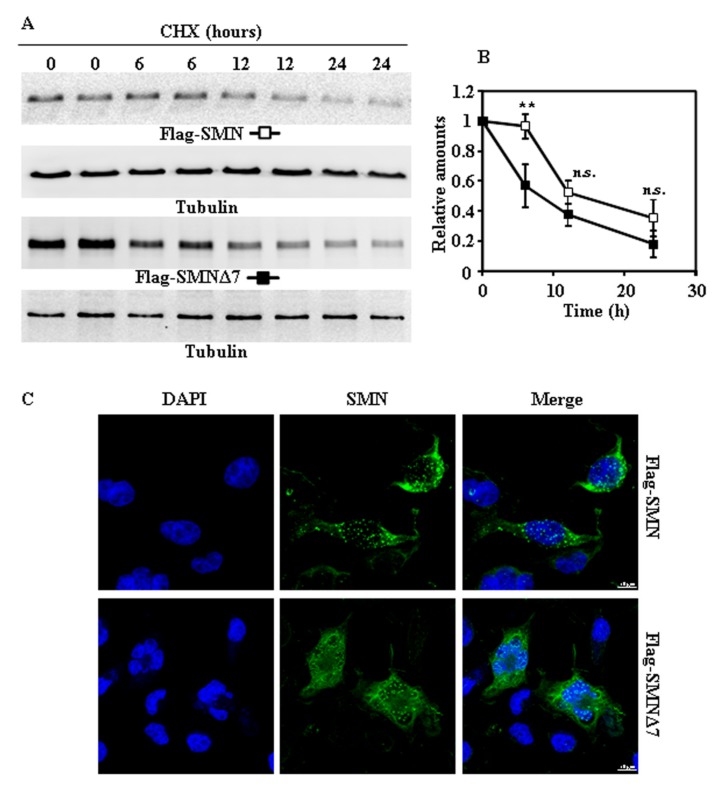
Degradation and subcellular localization of ectopically expressed Flag SMN and Flag-SMNΔ7 in HeLa cells. (**A**) HeLa cells were transiently transfected with SMN or SMNΔ7 tagged at the N-terminus with Flag and treated with CHX for the indicated times. Total cell lysates were analyzed by Western and immunoblot with anti-Flag specific antibodies; technical replicates are shown for each time point. Anti-tubulin antibodies were used as total protein loading control. (**B**) Graph shows the quantification of the proteins analyzed. (**C**) Confocal fluorescence localization of Flag-SMN or Flag-SMNΔ7 in transfected HeLa cells growing under basal conditions, analyzed by indirect immunofluorescence with anti-Flag specific antibodies (green channel) and counterstained for nuclei with DAPI (blue channel). Bars = 10 µm. Values are expressed as mean ± S.E.M. from three different experiments. Asterisks indicate a statistical significant difference between pairs (** *p* < 0.01, Student’s *t*-test). n.s., not significant.

**Figure 3:**
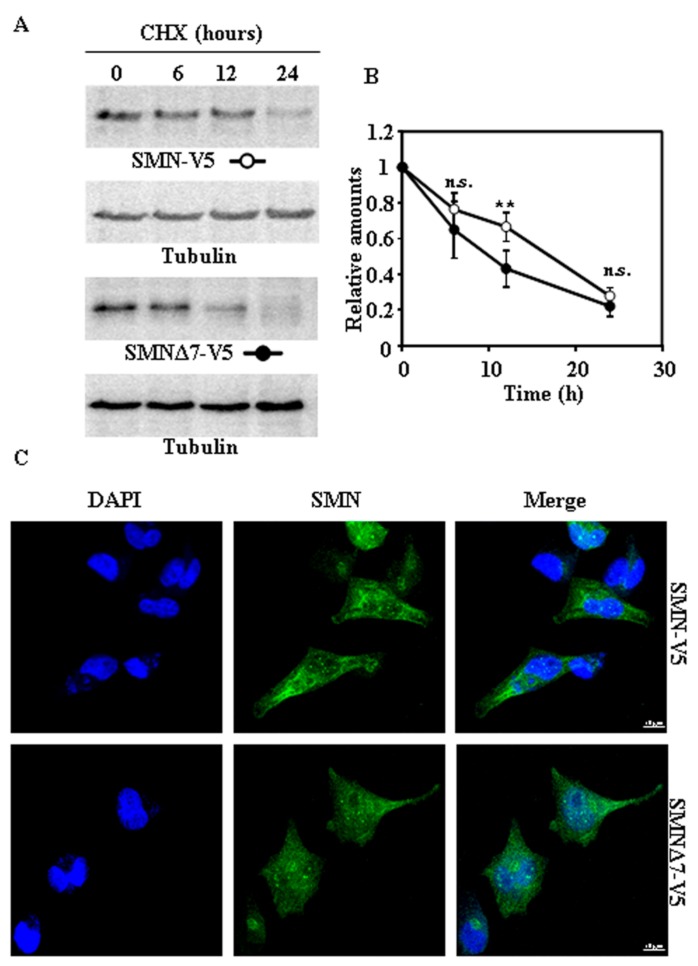
Degradation and subcellular localization of ectopically expressed SMN-V5 and SMNΔ7-V5 in HeLa cells. (**A**) HeLa cells were transiently transfected with SMN or SMNΔ7 tagged at the C-terminus with V5 and treated with CHX for the indicated times. Total cell lysates were analyzed by Western and immunoblot with anti-V5 specific antibodies. Anti-tubulin antibodies were used as total protein loading control. (**B**) Graph shows the quantification of protein levels referred to untreated cells as controls. (**C**) Confocal fluorescence images of SMN or SMNΔ7-V5 in transfected HeLa cells growing under basal conditions, analyzed by indirect immunofluorescence with anti-V5 specific antibodies (green channel) and counterstained with DAPI for nuclei visualization (blue channel). Bars = 10 µm. Values are expressed as mean ± S.E.M. from at least three different experiments. Asterisks indicate a statistical significant difference between pairs (** *p* < 0.01, Student’s *t*-test). n.s., not significant.

Taking into account that tags added to the N- or C-terminus of a protein may affect its degradation rates [12], we used SMN and SMNΔ7 with either Flag at the N-terminus or V5 tags at the C-terminus. Exogenously expressed Flag-SMN and Flag-SMNΔ7 (Figure 2A,B) proteins, after treatment of cells with CHX, showed marginal differences in degradation rates. Subcellular localization of both Flag-tagged SMN proteins (Figure 2C) showed a distribution similar to that found for endogenous SMN (Figure 1C), but with increased number of nuclear “foci” (Figure S1). Comparison of degradation of SMN and SMNΔ7 tagged with V5 at C-terminus (Figure 3A,B) showed only significant difference at 12 h after CHX addition. Again, the distribution in cells of both SMN and SMNΔ7 proteins tagged with V5 (Figure 3C) at the C-terminus was not substantially different to that found for endogenous SMN (Figure 1C). In contrast to N-terminal Flag-tagged versions of the proteins (Figure 2C), there was no increase in the number of “foci” in the nuclei (Figure S1). Comparison of the degradation rates of Flag (N-terminal) and V5 (C-terminal)-tagged SMN and SMNΔ7 (Figure S2) showed no significant difference, except for the earliest time point (6 h), where Flag-SMN degradation was smaller than SMN-V5. Those results showed that tagging SMN either at the N- or C-terminus did not significantly alter the rate of degradation of SMN and SMNΔ7, and SMNΔ7 constructs had a moderate, but significant, tendency to faster degradation than SMN.

### 2.3. Requirement of Ubiquitylation of SMN Proteins for Degradation

**Figure 4:**
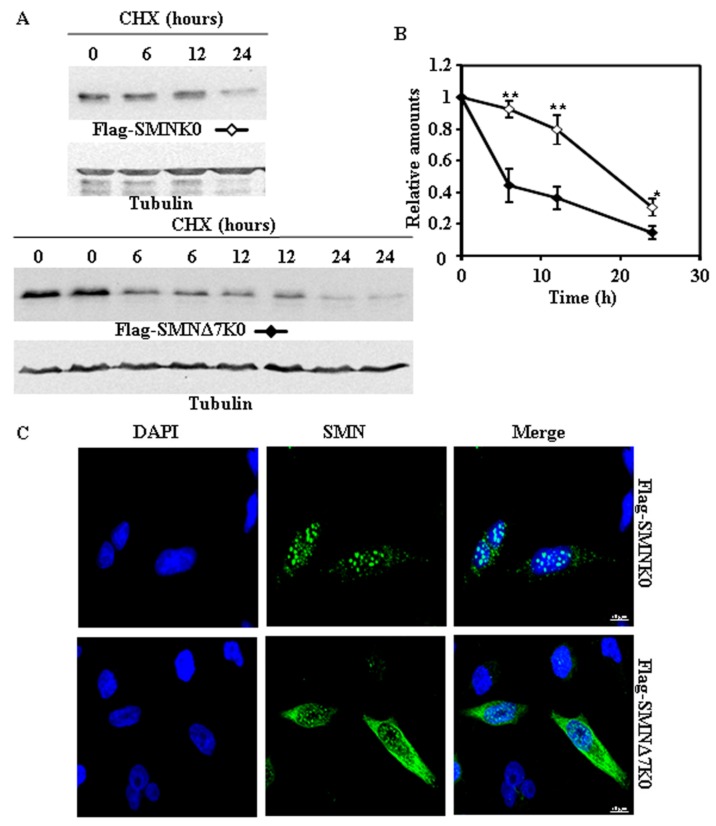
Degradation and subcellular localization of ectopically expressed Flag-SMNK0 and Flag-SMNΔ7K0 in HeLa cells. (**A**) HeLa cells were transiently transfected with SMNK0 or SMNΔ7K0 (replicates are shown for each time point) tagged at the N-terminus with Flag and treated with CHX for the indicated times. Total cell lysates were analyzed by Western and immunoblot with anti-Flag specific antibodies. Anti-tubulin antibodies were used as total protein loading control. (**B**) Graph of the quantification of the corresponding immunoblots. (**C**) Confocal fluorescence images of HeLa cells transfected with Flag-SMNK0 and Flag-SMNΔ7K0, stained with anti-FLAG specific antibodies (green channel) and counterstained for nuclei with DAPI (blue channel). Bars = 10 µm. Values are expressed as mean ± S.E.M. from three different experiments. Asterisks indicate a statistical significant difference between pairs (* *p* < 0.05, ** *p* < 0.01, Student’s *t*-test). n.s., not significant.

**Figure 5:**
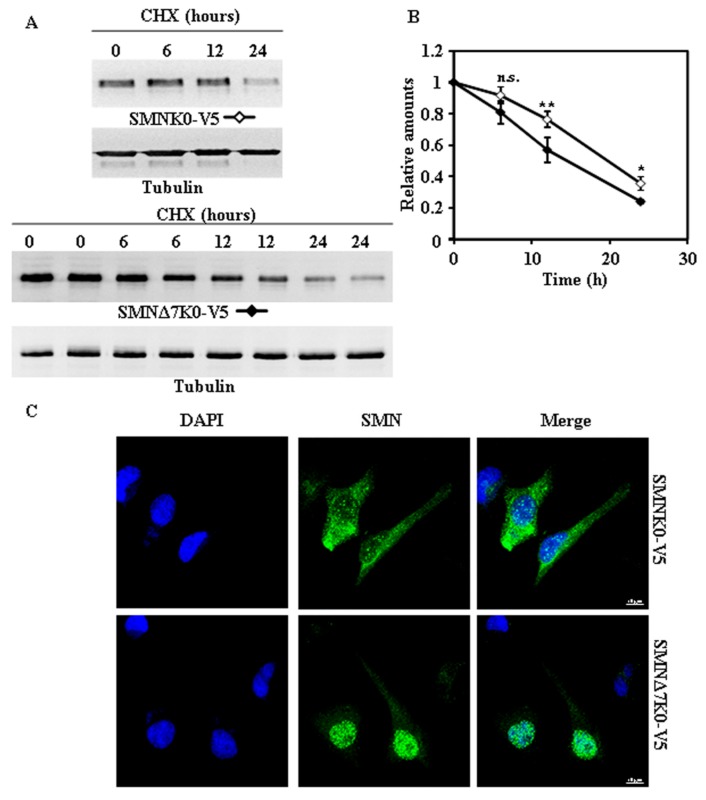
Degradation and subcellular localization of ectopically expressed SMNK0-V5 and SMNΔ7K0-V5 in HeLa cells. (**A**) HeLa cells were transiently transfected with SMNK0 or SMNΔ7K0 (replicates are shown for each time point) tagged at the C-terminus with V5 and treated with CHX for the indicated times. Total cell lysates were analyzed by Western and immunoblot with anti-V5 specific antibodies. Anti-tubulin antibodies were used as total protein loading control. (**B**) Graph of the quantification of the corresponding immunoblots. (**C**) Confocal fluorescence images of HeLa cells transfected with SMNK0-V5 and SMNΔ7K0-V5, stained with anti-V5 specific antibodies (green channel) and counterstained for nuclei with DAPI (blue channel). Bars = 10 µm. Values are expressed as mean ± S.E.M. from at least three different experiments. Asterisks indicate a statistical significant difference between pairs (* *p* < 0.05, ** *p* < 0.01, Student’s *t*-test). n.s., not significant.

Based on previous studies, we expected that degradation of SMN and SMNΔ7 would be dependent on prior ubiquitylation. To determine the Lys residues of SMN involved in the covalent ubiquitin modification of SMN, we searched public protein databases and found that SMN is ubiquitylated at amino acids K41, K51, K184, K186 and K190 by studies of cell ubiquitinome [13,14]. We selected the SMNΔ7 construct tagged with V5 at the C-terminus for the initial studies. Either a single substitution (K41R) or substitution of K41, K51, and K186 by R had no significant effect on the rate of degradation of SMNΔ7 tagged with V5 at the C-terminal. Accordingly, we made constructs of SMN and SMNΔ7 with substitution of all Lys residues by Arg (Lys-less or K0 variants). Figure 4 and Figure 5 show the degradation of K0 variants of SMN and SMNΔ7 either tagged at the N-terminus (Flag) or C-terminus (V5), respectively. SMN-K0, either tagged at the N-terminus (Figure 4A,B) or C-terminus (Figure 5A,B), showed a reduced rate of degradation compared to the corresponding SMNΔ7-K0, being more extensive in the case of N-terminal Flag-tagged constructs (Figure 4A,B) than for the V5 C-terminal tagged proteins (Figure 5A,B). A comparison of N-terminal (Flag) with C-terminal (V5) tagged K0 constructs, showed (Figure S3) no difference for SMN and only significant difference at the earlier time point (6 h) for the SMNΔ7 K0 constructs. Regarding subcellular localization, the N-terminal Flag tagged SMN K0 (Figure 4C) was almost exclusively localized in the cell nucleus, while C-terminal V5-tagged SMN K0 (Figure 5C) was localized both in the nucleus and cytoplasm, in both cases with increased number of nuclear “foci” (Figure S1). Similar results for the Flag-SMN K0 have been previously described [11]. In contrast, Flag-SMNΔ7 K0 was localized in the nucleus and cytoplasm (Figure 4C), and SMNΔ7 K0-V5 construct was localized almost exclusively in the cell nucleus (Figure 5C), again with an increase in the number of nuclear foci for these constructs (Figure S1).

**Figure 6:**
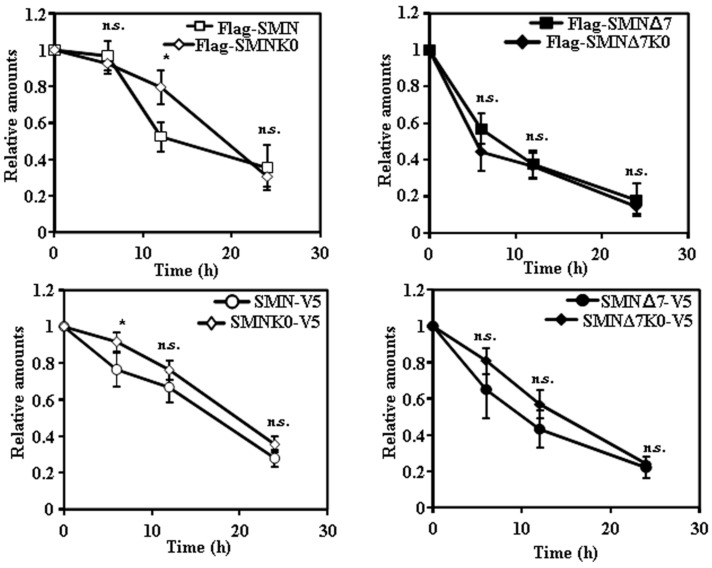
Comparisons of the time-course of degradation of SMN and SMNΔ7 tagged at the N- or C-terminus with the corresponding Lys-less (K0) mutants. The data for these plots are derived from previous results shown in Figure 2, Figure 3, Figure 4 and Figure 5. Asterisks indicate a statistical significant difference between pairs (* *p* < 0.05, Student’s *t*-test). n.s., not significant.

The final comparisons between the rates of degradations of the different wild-type SMN constructs with the K0 constructs are presented in Figure 6. From those re-plots of the data already shown in previous figures, it may be concluded that changing all Lys residues to Arg (Lys-less. K0 constructs) had marginal effect on the degradation of SMN and SMNΔ7 tagged either at the N or C-terminus.

### 2.4. Effect of Proteasome Inhibition on the Degradation of Endogenous SMN and Ectopically Expressed SMN Constructs

**Figure 7:**
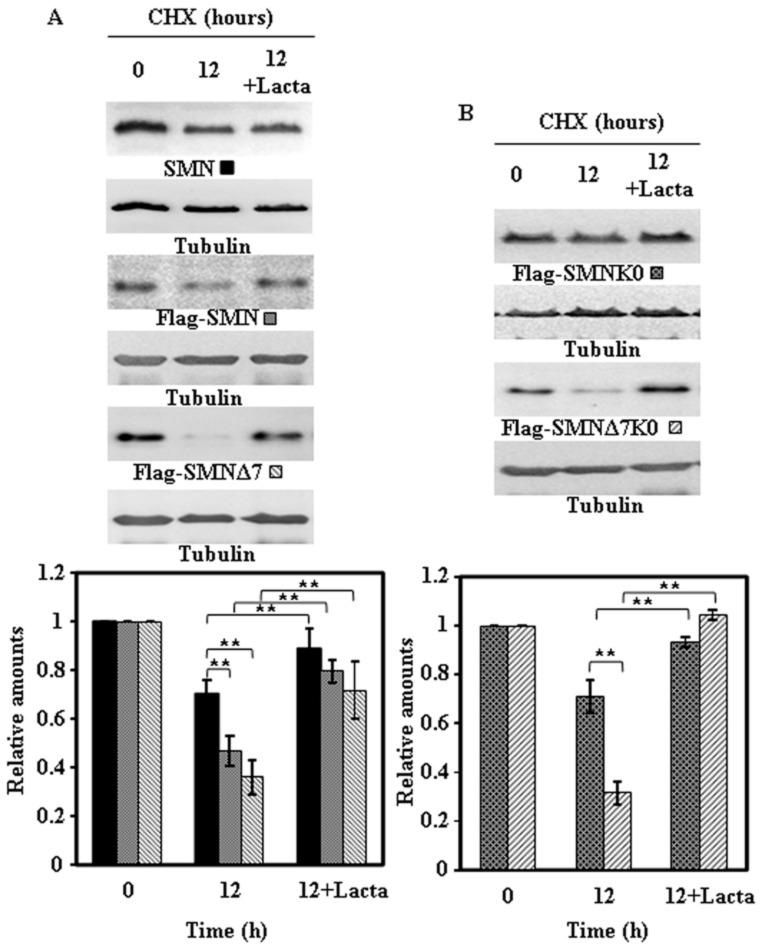
Effect of proteasome inhibition on the degradation of endogenous and ectopically expressed Ffag-SMN or Flag-SMNΔ7 constructs in HeLa cells. Untransfected (endogenous SMN) or transiently transfected HeLa cells with Flag-SMN or Flag-SMNΔ7 (**A**) and Flag-SMNK0 or Flag-SMNΔ7K0 (**B**) were treated with CHX in the absence or in the presence of Lactacystin (Lacta) for the indicated times and cell extracts were analyzed by immunoblot with SMN or anti-Flag specific antibodies. Anti-tubulin antibodies were used as total protein loading control. The graphs below show the quantification of the corresponding immunoblots. Values are expressed as mean ± S.E.M. from three different experiments. Asterisks indicate a statistical significant difference between pairs (** *p* < 0.01, Student’s *t*-test). n.s., not significant.

**Figure 8:**
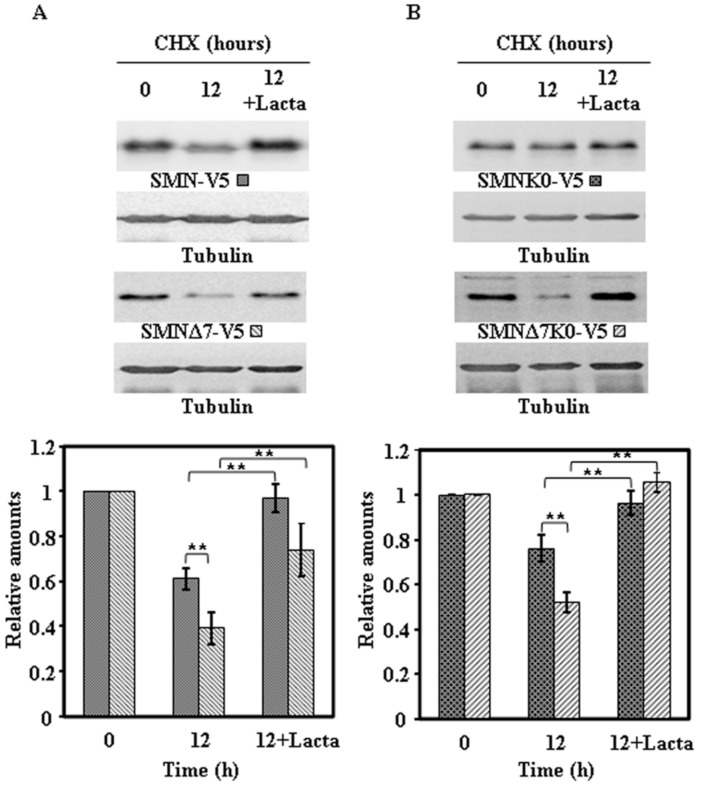
Effect of proteasome inhibition on the degradation of ectopically expressed SMN-V5 or SMNΔ7-V5 constructs in HeLa cells. Transiently transfected HeLa cells, with SMN-V5 or SMNΔ7-V5 (**A**) and SMNK0-V5 or SMNΔ7K0-V5 (**B**) were treated with CHX in the absence or in the presence of Lactacystin (Lacta) for the indicated times and cell extracts were analyzed by immunoblot with anti-V5 specific antibodies. Anti-tubulin antibodies were used as total protein loading control. The graphs below show the quantification of the corresponding immunoblots. Values are expressed as mean ± S.E.M. from three different experiments. Asterisks indicate a statistical significant difference between pairs (** *p* < 0.01, Student’s *t*-test). n.s., not significant.

To ascertain if the degradation of endogenous SMN and ectopically expressed proteins was due to the proteasomal pathway, similar experiments to the ones described above were performed in the absence or presence of lactacystin, an irreversible highly specific proteasome inhibitor. The results obtained for the endogenous SMN and the Flag-SMN constructs are presented in Figure 7 and those for the V5-tagged constructs in Figure 8: Clearly endogenous SMN, Flag-tagged (Figure 7A) and V5-tagged constructs (Figure 8A) and the corresponding K0 variants (Figure 7B and Figure 8B) degradation was inhibited by the presence of lactacystin.

These results showed very clearly that endogenous SMN and ectopically expressed SMN constructs are mainly degraded by the proteasomal pathway, with other possible pathways playing a minor role in SMN degradation.

## 3. Discussion

The results presented in this work show that endogenous SMN protein under steady-state conditions, while degraded by the proteasomal pathway has a half-life around 24 h in HeLa cells (Figure 1 and Figure 7). These results are in agreement (within a factor of 2) with those obtained by Stable Isotope Labeling by Aminoacids in Cell culture and Mass Spectrometry (SILAC-MS) experiments of global protein degradation showing an average of 47.3 h in NIH·3T3 [15], 28.7 h in HeLa cells [16] and 40 h for the smn-1 homologue in *C. elegans* [17]. Pulse-chase experiments with ^35^S-methionine/cysteine as described by Burnett el al. [5] in HEK293T show a fast degradation rate of the newly-synthesized endogenous SMN, similar to the newly synthesized N-terminal Myc-tag SMN (half-life 4.3 h), and faster for N-terminal Myc-SMNΔ7 (half-life 2.2 h). This discrepancy is not unusual, many newly synthesized proteins in the cell are subjected to fast degradation [18], which does not reflect the actual degradation from steady-state protein levels that we analyze here, also partially analyzed by Burnett el al. [5]. Indeed, many newly synthesized proteins in the cell are subjected to fast degradation [18], which clearly does not reflect the actual degradation rate of the steady-state cellular pool of these proteins. Actually, it has been estimated that 20–30% of the newly synthesized, or “young” proteins, are degraded in a very short-time, mainly by the ubiquitin–proteasome pathway [19]. These degraded “young” proteins contribute more peptides to the peptidome presented by HLA class I than “older” proteins (fully mature and assembled), as judged by SILAC-MS experiments [20]. The above conclusion is also supported by the circumstantial evidence that SMN spliced peptides, produced by proteasome catalyzed peptide splicing, are recently reported in HLA class I peptidome analysis [21]. These data indicate that “young” newly synthesized SMN protein and the “old” protein have different probabilities of being degraded, greater for the “young” (newly synthesized protein) than for the “old”. Following this reasoning, the ectopically expressed SMN protein constructs analyzed after 24 h of expression, as studied here, may have a higher probability of degradation than the “old” endogenous SMN protein, possibly accounting for the observed difference between the half-life of endogenous SMN and the ectopically expressed SMN constructs.

To investigate the degradation of SMN and SMNΔ7, different authors and ourselves, have used ectopic expression of tagged-protein constructs; and the levels of expression of the ectopically expressed proteins were similar to endogenous SMN protein levels. Published studies have mainly used N-terminal tagged protein constructs: Myc [5], HA [6] and Flag [9,11]. Even small tags and its localization at the N-terminal or C-terminal in a protein construct can affect the degradation of proteins [12]. As a consequence, we approached the degradation studies of SMN proteins by tagging either at the N-terminus (Flag) or C-terminus (V5); the same approach was used with mutant constructs where all Lys residues were changed to Arg (K0). One conclusion of the present work is that the degradation of SMN and SMNΔ7 either tagged at their N- or C-terminus did not absolutely require internal Lys ubiquitylation. An alternative possibility was the requirement of ubiquitylation of the N-terminus known to be relevant for the degradation of several proteins including p21/Waf1/Cip1, ERK3 [22], p19Arf [23], p16/Ink4a [24], Id1 [25], MyoD [26], PGC1a [27] and ataxin-3 [28]. The data reported here, and the results already published by other authors [5,6,9,11] unmentioned in their publications, showed that N-terminal tag of SMN and SMNΔ7, precluding N-terminal Met ubiquitylation, did not prevent degradation. These results discarded possible ubiquitylation of the N-terminal residue of the proteins as essential for SMN or SMNΔ7 degradation. We have also tried (by cotransfection of wild-type or K0 constructs with HA-ubiquitin) to detect both canonical and non-canonical ubiquitylation sites like Ser/Thr/Tyr or Cys [29], but no ubiquitylation was detected even in the presence of proteasome inhibitors. These negative results are in agreement with results already published; no ubiquitylation of SMN was detected unless an E3 ligase is overexpressed by cotransfection into cells [10,11]. Another critical point, overlooked previously, is that tagging at the N-terminus of a protein also blocks its N-terminal acetylation. In fact, the N-terminal of SMN (and likely of SMNΔ7) has been shown to be acetylated at two positions in acetylome studies [30]. The N-terminal sequence of SMN is M**A**M**S**S (acetylated residues indicated with bold characters). The acetylation observed is either at the N-terminal Ala2 residue, exposed after removal of the N-terminal Met by methionine aminopeptidase or at Ser4. Ser4 acetylation is likely due to a translational initiation of SMN protein at Met3, leaving Ser4 at the N-terminus after Met3 removal by the aminopeptidase [30]. Accordingly, SMN (and likely SMNΔ7) could be a substrate of the N-terminal acetylated N-rule pathway of degradation mediated by Doa10/March6 [31] in a physiological context and tagging at the N-terminus prevents the correct processing and N-terminal acetylation [31]. The precise role of N-terminal acetylation in SMN degradation and functionality remains to be established; here, we conclude that is not essential for degradation. It is also worth to mention that the results reported in this work are not in contradiction with the reported effect of E3 ligases Mib1 [10] and Itch1 [11] or USP9X [9] and UCHL1 [7] on SMN degradation. Certainly, both ubiquitin-dependent and -independent mechanisms (26S or 20S proteasome dependent) participate in the degradation of many proteins in the cell [32,33,34,35].

The general limitation of this data, extensive to other published studies using similar approaches, is that the mutant constructs, even if used untagged and in cells where the endogenous SMN has been interrupted, can only prove if a determined mechanism is necessary or essential for degradation, but not if it is relevant in normal cell physiology. Those constructs are unlikely to match the post-translational modifications and interactions of the natural cell endogenous protein. More than 40 different proteins are known to interact with SMN through different regions participating in snRNP and SnoRNP assembly, pre-mRNA splicing, actin transport and dynamics, etc. [36]. Lysines are specially enriched within the basic/lysine rich region of SMN, but they are also present in the Tudor and Y/G dimerization domains. While changing Lys to Arg may keep the charge, still the interactions with other proteins in those regions, as well as post-translational modifications of the mutant protein construct with respect to the wild type are likely to be affected [37]. Accordingly, to answer the question regarding the physiological mechanism of degradation of “old” SMN (present in the cell under steady-state conditions) requires another experimental approach. SMN, like many proteins in the cell, dimerizes, interacts with other cellular proteins forming macromolecular complexes, is subjected to post-translational modifications and is localized both in the cytoplasm and the nucleus [38]. The turnover of components of a protein complexes, like SMN or SMNΔ7, is dependent on many factors and regulatory mechanisms leading to the correct stoichiometry [39]. As mentioned above, quantitative proteomics and SILAC-MS experiments are well-suited for the study of bulk SMN (and other protein components of the SMN complexes) degradation. Nevertheless, those techniques are insufficient to untangle the problem of SMN turnover. There is a need of developing deep proteomic techniques to study the turn-over of the components of SMN (orphan and bound) complexes [39]. Those developments will allow a clear picture of the basic physiological mechanisms of degradation of steady state SMN protein in its different complexes and its regulation. Once this general picture is clear, in-depth proteomic studies using traditional mutational approaches can be applied. Eventually, those studies will allow the design of strategies aimed to increase SMN expression levels through regulation of the degradation as a possible therapeutic intervention for SMA patients [40].

## 4. Materials and Methods

### 4.1. DNA Constructs

DNA constructs for expression of SMN (human SMN) and SMNΔ7 (human SMNΔ7) were generated from human SMN1 cDNA cloned into pDNR-LIB vector (SourceBioscience, Nottingham, UK) (clone ID IMAGE 4250429, MGC 72037, BC062723) by PCR (4 min, 97 °C; 30 cycles of 45 s, 95 °C; 1 min, 59 °C and 1.5 min, 72 °C, and a final polymerization cycle of 15 min, 72 °C) using the following oligonucleotides: forward BamH1 SMN, 5′-GACGGATCCCATATGGCGATGAGC-3′ and reverse XhoI SMN 5′-GCGCTCGAGTTAATTTAAGGAATGTGAGCACC-3′. The same forward BamHI SMN and the reverse XhoI non-stop hSMN: 5′-GCGCTCGAGGCTAAATTTAAGGAATGTGAGCACC-3′. Amplified DNA was subcloned into pcDNA 3.1/V5-HisTopo TA vector (Invitrogen Waltham, MA, USA) after digestion with BamHI/XhoI. SMN2∆7 was generated by PCR amplification with the same template and the forward primer BamH1 SMN as above and the reverse primer XhoI SMN2Δ7: 5′-GCGCTCGAGCTATGCCAGCATTTCCATATAATAGCCAGTATGATAGCCACTCA-3′ or the reverse XhoI non-stop: SMN2Δ7 5′-GCGCTCGAGGCCAATGCCAGCATTTCCATATAATAGCCAGTATGATAGCCACTCA-3′ and inserted after digestion with BamH1/XhoI into pcDNA 3.1/V5-HisTopo TA vector digested with the same restriction enzymes. N-terminal Flag-tagged SMN or SMNΔ7 constructs were generated by PCR using the following oligonucleotides: forward BamHI Flag-SMN 5′ GACGGATCCATGGACTATAAGGACGATGATGACAAGATGGCGATGAGC 3′ and the reverse XhoI SMN as above or the same forward BamHI Flag-SMN and the reverse primer XhoI SMN2Δ7 already described. Amplified DNAs were subcloned into pcDNA3.1 vector (Invitrogen) after digestion with BamHI/XhoI. Lysine-less variants (K0) with all Lys residues changed to Arg of SMN or SMNΔ7 were synthesized by GenScript and subcloned into the appropriate vectors as indicated above. The DNA sequence of all constructs was verified by Sanger sequencing in an ABI Prism 3130XL.

### 4.2. Studies of Endogenous and Ectopically Expressed SMN Protein Degradation

HeLa cells were grown in Dulbecco’s modified Eagle’s medium (DMEM, Gibco BRL, Waltham, MA, USA) supplemented with 10% foetal bovine serum (Sigma-Aldrich, St. Louis, MO, USA) and 100 µg/mL gentamycin, at 37 °C and 5% CO_2_. For transfections, cells were plated at 3 × 10^5^ cells/well in 6-well plates and transfected with Lipofectamine 2000 (Invitrogen). Transfected HeLa cells were plated 24 h after transfection on 60 mm dishes and treated with the protein synthesis inhibitor cycloheximide (CHX, 25 μg/mL, Sigma, St. Louis, MO, USA) for the times indicated, in the absence or in the presence of 20 µM Lactacystin (proteasome inhibitor, St. Louis, MO, USA) as indicated. Cells viability after the different treatments (up to 24 h) was >95% as judged by Trypan-blue exclusion. After the treatments, cells were washed with cold phosphate-buffered saline (PBS) and directly lysed in SDS-buffer (62.5 mM Tris-HCl pH 6.8, 2% SDS, 5 mM DTT, 20% glycerol, 10 μM leupeptin, 1 μg/mL pepstatin and 1 mM PMSF). Cell extracts were sonicated for 10 min on ice, centrifuged at 14,000× *g* for 30 min and supernatants used to measure total protein concentration with BCA protein assay kit (Thermo Scientific-Pierce, Waltham, MA, USA). Total proteins (20 µg) were separated onto 12% SDS-PAGE gels and transferred to nitrocellulose or PVDF membranes for Western immunoblot analysis. Membranes were blocked with TTBS (50 mM Tris-HCl pH 7.5, 150 mM NaCl, 0.1% Tween-20) with 3% BSA overnight. The blots were then probed with anti-SMN1 antibodies at 1: 1000 (clone 8/SMN from BD Transduction; Franklin lakes, NJ, USA), anti-Flag (Sigma) at 1: 5000 or anti-V5 antibodies (Invitrogen) at 1:5000 as indicated. Anti α-Tubulin (DM1A, Sigma, dil. 1:5000) monoclonal antibody was used as total protein loading control. Blots were developed with species-specific antisera conjugated with horseradish peroxidase (1:5000, BioRad, Hercules, CA, USA) and detected with a chemiluminiscent method (MF-ChemiBIS 3.2, DNR Bio-Imaging Systems; Neve Yamin, Israel). Blots were analyzed by quantitative densitometry using Totallab TL100 software (version 1.0, TotalLab Ltd., Newcastle upon Tyne, UK). Results are expressed as mean ± S.E.M. for a minimal number of three independent experiments.

### 4.3. Immunofluorescence and Confocal Microscopy

Cells grown on coverslips were washed 3 times with cold PBS (20 mM Phosphate buffer pH 7.4, 137 mM NaCl, 2.7 mM KCl) fixed with 4% paraformaldehyde in PBS for 20 min at room temperature and permeabilized and blocked with 0.1% Triton X-100 containing 3% BSA in PBS for 30 min at room temperature. Primary antibodies (mouse anti-SMN at 1:300, mouse anti-V-5 at 1:200 or mouse anti-FLAG at 1:200) were added in blocking solution without Triton X-100, and incubated 3 h at room temperature. After washing 3 times with PBS, coverslips were incubated with Alexa Fluor 488 conjugated anti-mouse antibody (1:1000 dilution) and 4′,6-diamidino-2-phenylindole (DAPI, 5 µg/mL) for 1 h, washed again three with PBS. The coverslips were mounted with ProLong Gold antifade reagent for confocal microscopy observation in a laser scanning microscope (Leica TCS SP5). Images were captured with the same settings for all experiments presented. Under these settings no autofluorescence was detected. Controls, omission of primary or secondary antibodies, revealed no fluorescence. Images were processed using ImageJ software [41]. Quantification of nuclear *foci* in fluorescence images was analyzed with ImageJ 1.37c software [41] values reported as mean ± S.E.M. from an average of 45 cells per transfected SMN construct under study.

## 5. Conclusions

Taken together, these data show that SMN and SMNΔ7 proteasomal degradation does not absolutely require internal ubiquitylation nor N-terminal ubiquitylation or acetylation (prevented by N-terminal tagging). Nevertheless, we rationalize the need of deep proteomic techniques for the analysis of the turn-over of SMN complex components (orphan and bound) to precisely determine the physiological mechanisms of degradation of SMN and SMNΔ7 in the cell.

## Supplementary Materials

Supplementary materials can be found at www.mdpi.com/1422-0067/18/12/2667/s1.

## Abbreviations

CHX	Cycloheximide
DAPI	2-(4-Amidinophenyl)-6-indolecarbamidine dihydrochloride, 4′,6-Diamidino-2-phenylindole dihydrochloride
PMSF	Phenylmethanesulfonyl fluoride
PVDF	Polyvinylidene difluoride
SMA	Spinal Muscular Atrophy
