# Supplementary material for: Lysine-Less Variants of Spinal Muscular Atrophy SMN and SMNΔ7 Proteins Are Degraded by the Proteasome Pathway
Source: Int J Mol Sci. 2017 Dec 8;18(12):2667. doi: 10.3390/ijms18122667 (PMC5751269; doi:10.3390/ijms18122667)
Supplement: Supplementary file 1 [file ijms-18-02667-s001.pdf]

Supporting Information

Lysine-less variants of spinal muscular atrophy SMN and SMNΔ7 proteins are degraded by the proteasome pathway

Raúl Sánchez-Lanzas[1] and José G. Castaño[1]*.

[1]Departamento de Bioquímica, Instituto de Investigaciones Biomédicas "Alberto Sols". (UAM-CSIC), Centro de Investigación Biomédica en Red sobre Enfermedades Neurodegenerativas (CIBERNED). Facultad de Medicina UAM. 28029 Madrid

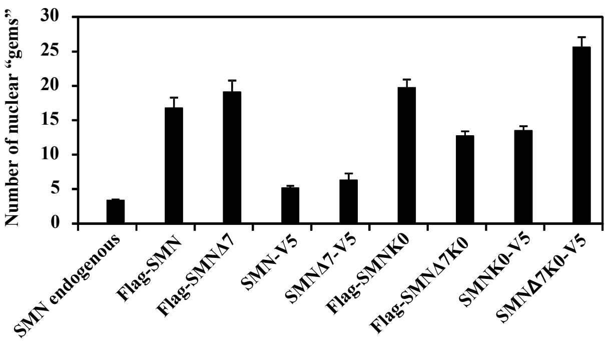

Supplementary Figure S1. Quantification of nuclear *foci* of SMN proteins. Graph shows the quantification of the number of nuclear *foci* per cell in HeLa cells not transfected, endogenous SMN (Endo. SMN), or transfected with the indicated SMN protein construct. Quantification of the confocal fluorescence images from two different transfection experiments in at least 45 cells for each construct were analyzed and data shows the average number of foci found per cell as mean ± S. E. M.

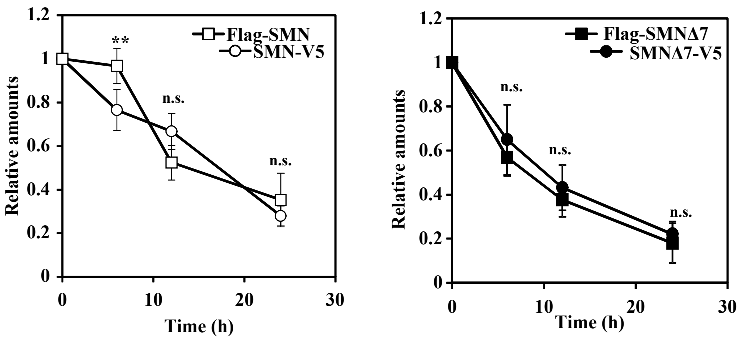

Supplementary Figure S2. Comparisons of the time-course of degradation of SMN and SMNΔ7 tagged at the N- or C-terminus. The data for these plots are derived from previous results shown in Figs. 2 and 3. Asterisks indicate a statistical significant difference between pairs (** $p < 0.01$, Student's t-test). n.s., not significant.

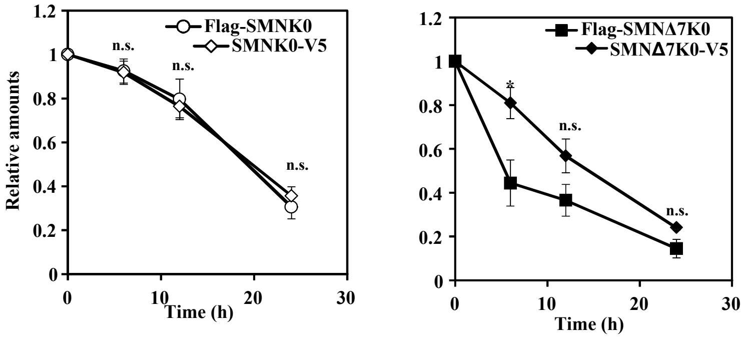

Supplementary Figure S3. Comparisons of the time-course of degradation of SMN and SMNΔ7 Lys-less (K0) mutants tagged at the N- or C-terminus. The data for these plots are derived from previous results shown in Figs. 4 and 5. Asterisks indicate a statistical significant difference between pairs (* $p < 0.05$, Student's t-test). n.s., not significant.
